# Impact of the Flavonoid Quercetin on β-Amyloid Aggregation Revealed by Intrinsic Fluorescence

**DOI:** 10.1021/acs.jpcb.2c02763

**Published:** 2022-09-19

**Authors:** Abeer Alghamdi, David J.S. Birch, Vladislav Vyshemirsky, Olaf J. Rolinski

**Affiliations:** † Photophysics Group, Centre for Molecular Nanometrology, Department of Physics, Scottish Universities Physics Alliance, University of Strathclyde, 107 Rottenrow East, Glasgow G4 0NG, United Kingdom; ‡ School of Mathematics and Statistics, University of Glasgow, Glasgow G12 8QQ, United Kingdom

## Abstract

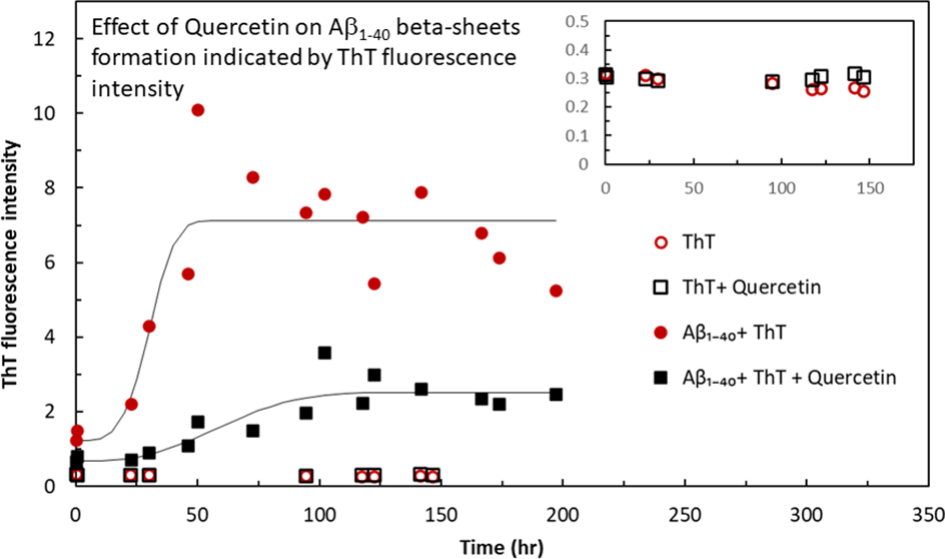

We report the effects of quercetin, a flavonoid present in the human diet, on early stage beta-amyloid (Aβ) aggregation, a seminal event in Alzheimer’s disease. Molecular level changes in Aβ arrangements are monitored by time-resolved emission spectral (TRES) measurements of the fluorescence of Aβ’s single tyrosine intrinsic fluorophore (Tyr). The results suggest that quercetin binds β-amyloid oligomers at early stages of their aggregation, which leads to the formation of modified oligomers and hinders the creation of β-sheet structures, potentially preventing the onset of Alzheimer’s disease.

## Introduction

Flavonoids consist of a large group of polyphenolic compounds having a benzo-γ-pyrone structure and are ubiquitous in plants. To date, more than 8000 varieties of flavonoids have been identified.^1,2^ One important subclass of flavonoids, the flavonols and their major representative quercetin, are the most prevalent flavonoids in the human diet.^3−5^ They occur in many vegetables and fruits such as onions, curly kale, broccoli, blueberries, and apples as well as in red wine, tea, and cocoa.

Quercetin has gained much scientific attention due to its antioxidant and metal ion-chelating properties^6−9^ and its capacity to inhibit amyloid fibril formation.^10^ Many human neurodegenerative diseases such as Alzheimer’s and Parkinson’s disease are associated with amyloid fibril formation; thus, quercetin is intensively researched for its therapeutic potential in providing improved treatment for neurodegenerative diseases.

Alzheimer’s disease (AD) is the most common progressive neurodegenerative disease and one of the leading causes of dementia globally.^11^ The two major pathological hallmarks of AD are extracellular β-amyloid (Aβ) plaques and intracellular τ-containing neurofibrillary tangles. The prevailing view of AD pathogenesis today^12−17^ is that accumulation of Aβ either as oligomers or fibrils initiates a pathophysiological cascade resulting in τ misfolding and aggregation that spreads throughout the brain, eventually leading to neural system failure and cognitive decline. This clearly implicates Aβ as a critical disease initiator. Consequently, most of the attention in the scientific community has focused on ways to reduce Aβ production, inhibit aggregation, increase removal, and identify the toxic amyloid forms.

**Figure 1:**
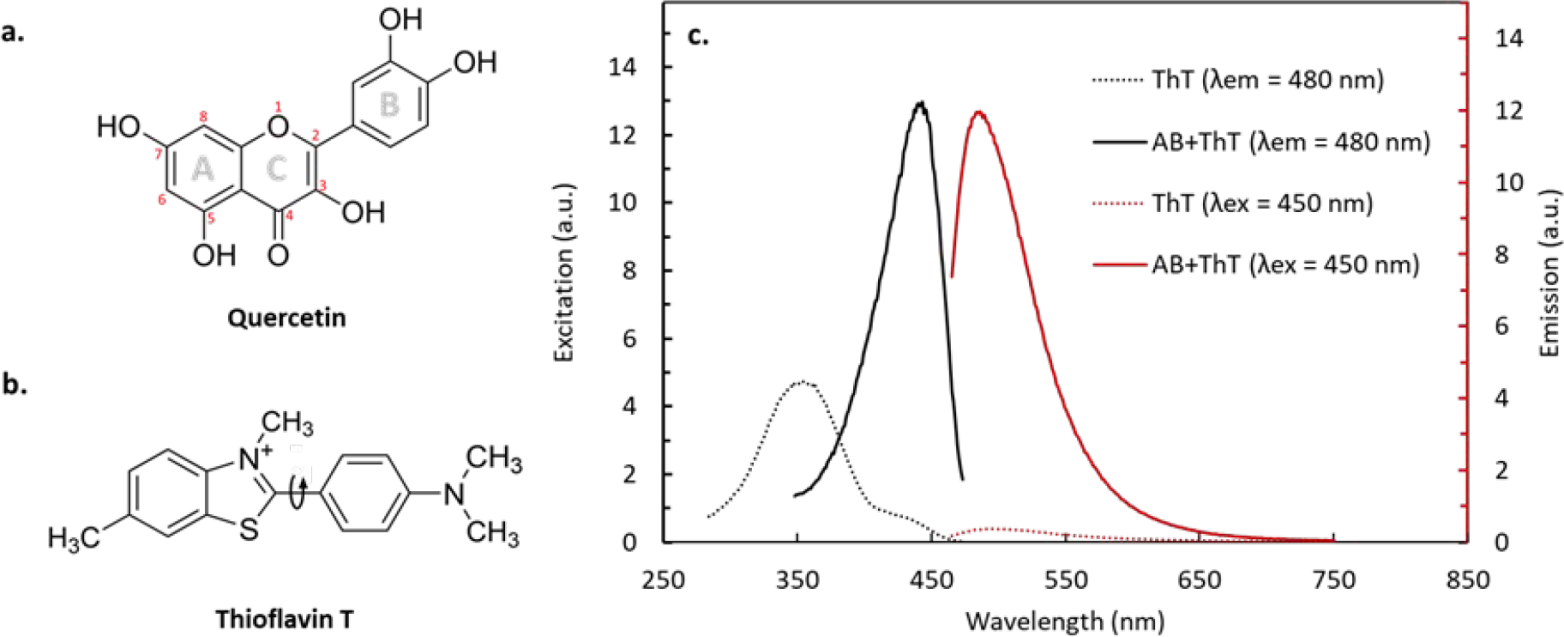
Chemical structure of quercetin (a) and thioflavin T (b). Fluorescence excitation (black) and emission (red) spectra of equal concentrations of ThT alone in HEPES buffer (dotted line) and ThT with Aβ_1–40_ in the same buffer (solid line) measured after 72 h of incubation (c).

Quercetin has been reported to exert antioxidant activity due to the catechol group in the β ring and the OH group located in positions 3 and 5 of the AC ring^18^ (Figure 1a). It is also suggested that quercetin might indirectly inhibit the formation of Aβ peptides by constraining the activity of the β-site APP cleaving enzyme (BACE-1).^19^ Studies have shown that OH groups and phenolic rings in flavonoids are essential for the noncovalent interactions with β-sheet structures, which are common to all amyloid proteins.^20^ Studies on quercetin in particular have pointed out that it has the potential to inhibit Aβ aggregation by forming hydrophobic interactions and hydrogen bonds with the formed β-sheets.^19^ More interestingly, it has been reported that quercetin has the ability to destabilize preformed fibrils in some proteins such as bovine insulin,^21^ α-synuclein,^22^ and Aβ_25–35_^23^ in a dose-dependent manner whereupon fibrils are transformed into amorphous aggregates.^21^ The final morphologies of protein assemblies in the presence of quercetin are different and require further examination for better characterization.

In this research, we have used the intrinsic fluorescence of Aβ_1–40_ peptides to study their oligomerization under the influence of quercetin. For a broader and more comprehensive understanding of the changes observed in the intrinsic Tyr_10_ fluorescence intensity decay, we measured time-resolved emission spectra (TRES), which can sufficiently demonstrate structural changes in Aβ_1–40_, including aggregation, as we have shown in previous studies.^24^

To identify the direct effects of quercetin on Tyr_10_ in Aβ_1–40_, which can be observed when the aggregation does not occur, we extended the lifetime measurements to the shorter fragments of the Aβ_1–40_ peptide, such as Aβ_1–11_ and Aβ_1–16_. These peptides also contain the single Tyr residue but lack the hydrophobic C-terminal of Aβ_1–40_, which is critical in triggering the transformation from the α-helical to β-sheet structure and plays a key role in aggregation.^25^

Further stages of Aβ_1–40_ fibril formation followed by oligomerization were monitored by the thioflavin T (ThT) binding assay (Figure 1b). ThT is currently considered the gold-standard fluorescent probe for the study of amyloid fibril formation. Upon binding to β-sheet-rich structures, ThT gives a strong fluorescence signal at 482 nm when excited at 450 nm. The mechanism by which ThT fluorescence is enhanced upon binding to amyloids has been ascribed to the rotational immobilization of the C–C bond between the benzothiazole and aniline rings^26,27^ (Figure 1b), which results in a dramatic shift in the ThT excitation maximum from 350 to 450 nm as shown in Figure 1c. Although ThT is an efficient reporter of fibril formation, its poor photophysical and binding properties make it ill-suited for detection of the small oligomeric species^28^ that are critical precursors to fibril formation. Thus, the ThT assay is used here as a complementary technique, to confirm the formation of Aβ_1–40_ fibrils.

## Experimental Section

### Sample Preparation

The lyophilized Aβ_1–40_ powder (Aβ_1–40_; Sigma-Aldrich, UK) was treated before use with 1,1,1,3,3,3- hexafluoro-2-propanol (HFIP; Sigma-Aldrich, UK). This procedure is routinely used^29,30^ to ensure that the starting sample comprises only monomers. Aβ_1–40_ was diluted in 100% HFIP to 0.1 mM and sonicated for 10 min. The clear solution containing the dissolved peptide was then aliquoted in Eppendorf microcentrifuge tubes, and the HFIP was allowed to evaporate in a fume hood. The samples were then stored at −20 °C.

Upon usage, the film of Aβ_1–40_ was dissolved in 1% NH4OH at a concentration of 1 mg/mL, and this was sonicated for 30 s to 1 min. To prepare the free Aβ_1–40_ sample, the solution was diluted in 4-(2-hydroxyethyl)-1-piperazineethanesulfonic acid (HEPES; Sigma-Aldrich, UK, pH 7.4, 0.1 M) to a concentration of 50 μM. To prepare Aβ_1–40_ samples with quercetin, a HEPES buffer containing quercetin dihydrate (C_15_H_10_O_7_·2H_2_O; Riedel-de Haen, Germany) was added. The final concentations of components in the sample were 50 μM Aβ_1–40_ and 15 or 50 μM quercetin. For the ThT assay, a HEPES buffer containing ThT (Sigma-Aldrich, UK) with and without quercetin was added. The final concentrations of components in the sample were 50 μM Aβ_1–40_, 15 μM ThT, and 15 or 50 μM quercetin. All Aβ_1–40_ samples were incubated at a temperature of 36 °C over the entire time of the experiment.

Aβ_1–11_ and Aβ_1–16_ samples were prepared by dissolving Aβ_1–11_ and Aβ_1–16_ peptides (Aβ_1–11_/Aβ_1–16_; Sigma-Aldrich, UK) in 0.1 M HEPES to a concentration of 50 μM. Different concentrations of quercetin dihydrate (concentrations from 0 to 200 μM) were added to the solutions.

### Steady-State Measurements

Steady-state fluorescence spectra of ThT and tyrosine in Aβ_1–11,_ Aβ_1–16_, and Aβ_1–40_ were obtained using a Fluorolog-3 spectrofluorimeter. The excitation and emission monochromators were set at 5 nm slit widths. Tyr was excited at 279 nm, and emission spectra were recorded at 290–500 nm in 1 nm increments. Measurements were repeated for the Aβ_1–40_ sample at different times: 1, 24, 50/72, and 140 h after sample preparation. ThT was excited at 450 nm, and the emission spectra were recorded from 465 to 600 nm over a 200 h time period.

### Time-Correlated Single Photon Counting (TCSPC)

TCSPC measurements were conducted on a HORIBA Scientific DeltaFlex fluorometer (HORIBA Jobin Yvon IBH Ltd., Glasgow, UK). The system was equipped with Seya-Namioka monochromators with a focal length of 100 mm and a peak transmission efficiency of 62% for excitation and emission. Typical spectral bandwidths were 16 nm. The DeltaFlex system uses a HORIBA PPD photon counting detector (PPD-650), and a HORIBA NanoLED for excitation with a center wavelength of 279 nm, a pulse duration of 50 ps, and a repetition rate of 1 MHz.^31^

Fluorescence decay curves were obtained for Aβ_1–11_ and Aβ_1–16_ in the absence and presence of different concentrations of quercetin (25, 50, and 200 μM) at an emission wavelength of 316 nm.

For the Aβ_1–40_ TRES measurements, a series of 12 fluorescence decay curves were collected at the emission wavelengths between 297 and 330 at 3 nm increments. Figure S1 in Supporting Information (SI) shows an example of three fluorescence decays measured at different stages of aggregation (1, 24, and 140 h) for free Aβ_1–40_ and Aβ_1–40_ with quercetin at concentrations 15 and 50 μM.

Fluorescence decay curves were analyzed using DAS6 reconvolution software that assumes that the experimental curve *F*(*t*) directly accounts for the presence of scattered excitation light in the Tyr decay^32^
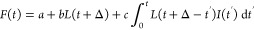
1where *L*(*t*) is the prompt excitation function, and *a*, *b*, and *c* are the background noise level, contribution of the scattered light, and the scaling parameter, respectively. Δ approximates the effect of the time-shift between the prompt and decay curves due to the transit time wavelength dependence of the photomultiplier detector. *I*(*t*) is the *n*-exponential model function. The recovered parameters of *I*(*t*) were used in the TRES analysis.

### Calculating Time-Resolved Emission Spectra (TRES)

The reason why we decided to use the TRES approach, instead of applying the fluorescence intensity decay analysis based on a measurement at a single wavelength, was the previously observed^24,33^ complexity of the fluorescence kinetics of Aβ_1–40_, where the decay of the population of excited molecules is combined with the shifts in the transient emission spectra caused by dielectric relaxation. The TRES approach allows the resolution of these two processes. The fluorescence decays *I*_*λ*_(*t*), measured at individual wavelengths λ (see Figure S1 in SI), were fitted to multiexponential functions and then used to calculate the TRES *I*_*t*_(*λ*) from the equation
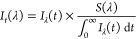
2where *S*(*λ*) is the steady-state fluorescence spectrum of the sample, and the integral is proportional to the total emitted photons in the lifetime experiment. The obtained spectra were then converted from the wavelength *I*_*t*_(λ) to the wavenumber scale according to *I*_*t*_(*ν*) = λ^*2*^*I*_*t*_(*λ*).

TRES were obtained for Aβ_1–40_ at several stages of aggregation, namely, 1, 24, 50 or 72, and 140 h after sample preparation (the number indicates the age of the sample when the measurement at the first wavelength has been started). From the data obtained for each stage of aggregation, 14 TRES were calculated at different times after excitation. Figure S2 shows the TRES of samples in the absence and presence of 15 and 50 μM quercetin at different stages of aggregation (i.e., 1, 24, 50 or 72, and 140 h).

According to Toptygin and Brand,^34^ the fluorescence spectrum of a single fluorescent residue can be expressed as *∼ ν*^3^*g*(*ν*), where *g*(*ν*) is the Gaussian distribution function. Therefore, for the purpose of detailed analysis of the *Aβ*_1–40_ TRES, we modeled the recovered spectra *I*_*t*_(*ν*) at the time *t* as the sum of *N* components of the type *∼ν*^*3*^*g*(*ν*)
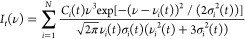
3where *t* is the time after excitation in ns, ν is the wavenumber in cm^–1^, *σ*_*i*_(*t*) is the standard deviation of each component, *ν*_*i*_(*t*) is its peak position, and *C*_*i*_(*t*) is the fluorescence intensity contribution of the *i*th component at the time *t*, i.e., the fluorescence intensity decay of this component.

The obtained experimental TRES for the sample with and without quercetin demonstrated the presence of two components (*N* = 2). The parameters recovered from fitting the proposed model (eq 3 for *N* = 2) to the experimental data were used to analyze and compare the fluorescence kinetics of both samples.

In the presence of dielectric relaxation, *ν*_*i*_(*t*) decays exponentially from a great value to a minimum value. To obtain the time of relaxation (τ_R_), *ν*_*i*_(*t*) decays were fitted to the equation
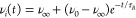
where ν_0_ and ν_∞_ are the position of the peak at *t* = 0 and *t* = ∞, respectively.

## Results and Discussion

### Aβ_1–11_ and Aβ_1–16_ Samples

**Figure 2:**
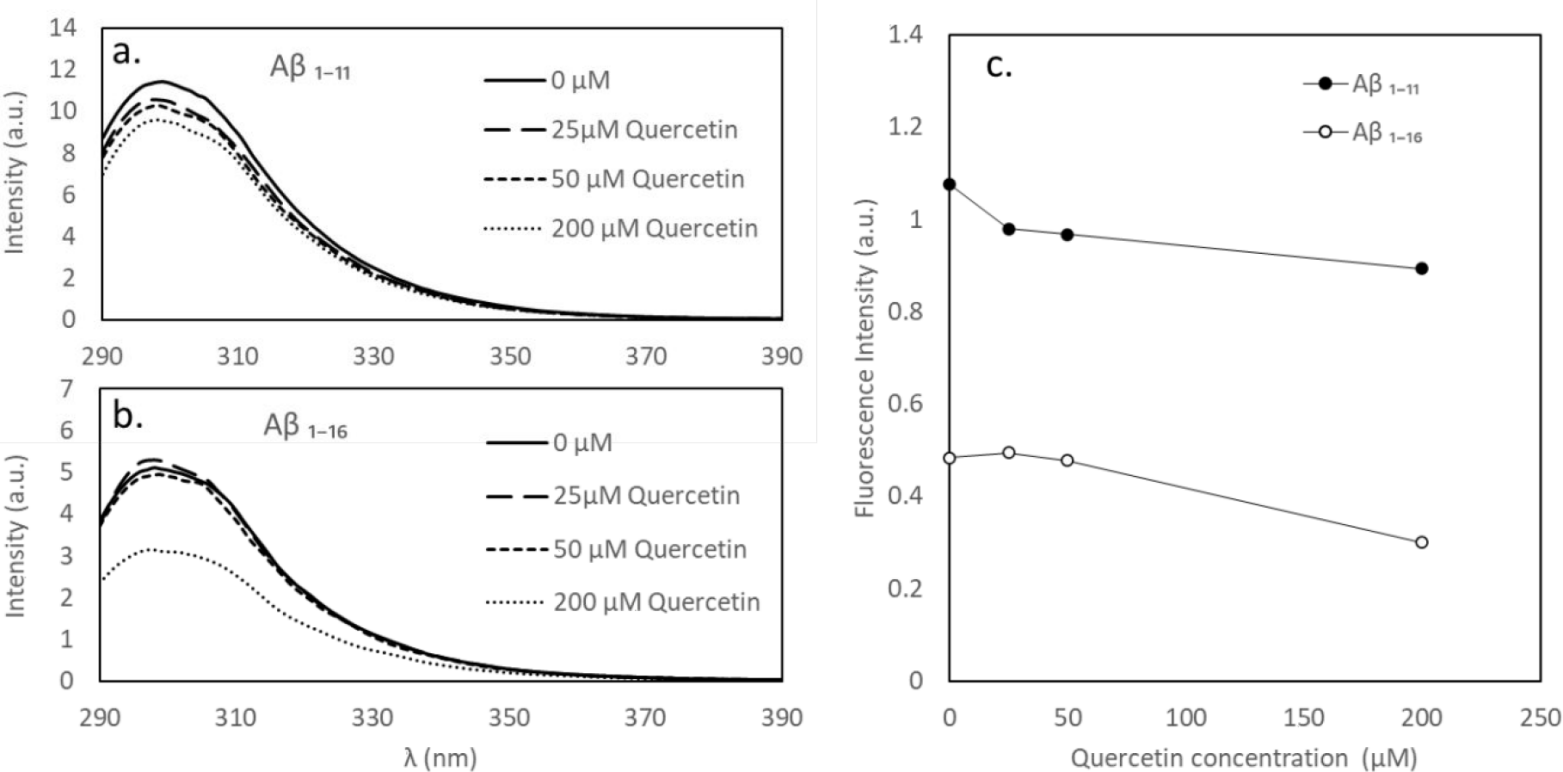
Emission spectra of Aβ_1–11_ (a) and Aβ_1–16_ (b) at excitation wavelength 279 nm in the absence (solid line) and presence of 25 μM (long dash), 50 μM (short dash), and 200 μM (dotted) quercetin. Maximum fluorescence intensity of Aβ_1–11_ (black circle) and Aβ_1–16_ (white circle) as a function of quercetin concentration (c).

Steady-state results show that increasing the concentration of quercetin up to 200 μM in a 50 μM Aβ_1–11_ sample slightly reduces the intensity of Tyr emission (Figure 2a). The emission of the 50 μM Aβ_1–16_ sample, on the other hand, remains relatively constant with an increase in quercetin concentration from 0 to 50 μM. However, at a high concentration of quercetin, 200 μM, the emission of Aβ_1–16_ is considerably reduced (Figure 2b). The comparison of changes in the peak intensities for both Aβ fragments (Figure 2c) shows a gradual drop with an increase in quercetin concentration, suggesting inner filter effects due to quercetin absorption (Figure S3).

**Figure 3:**
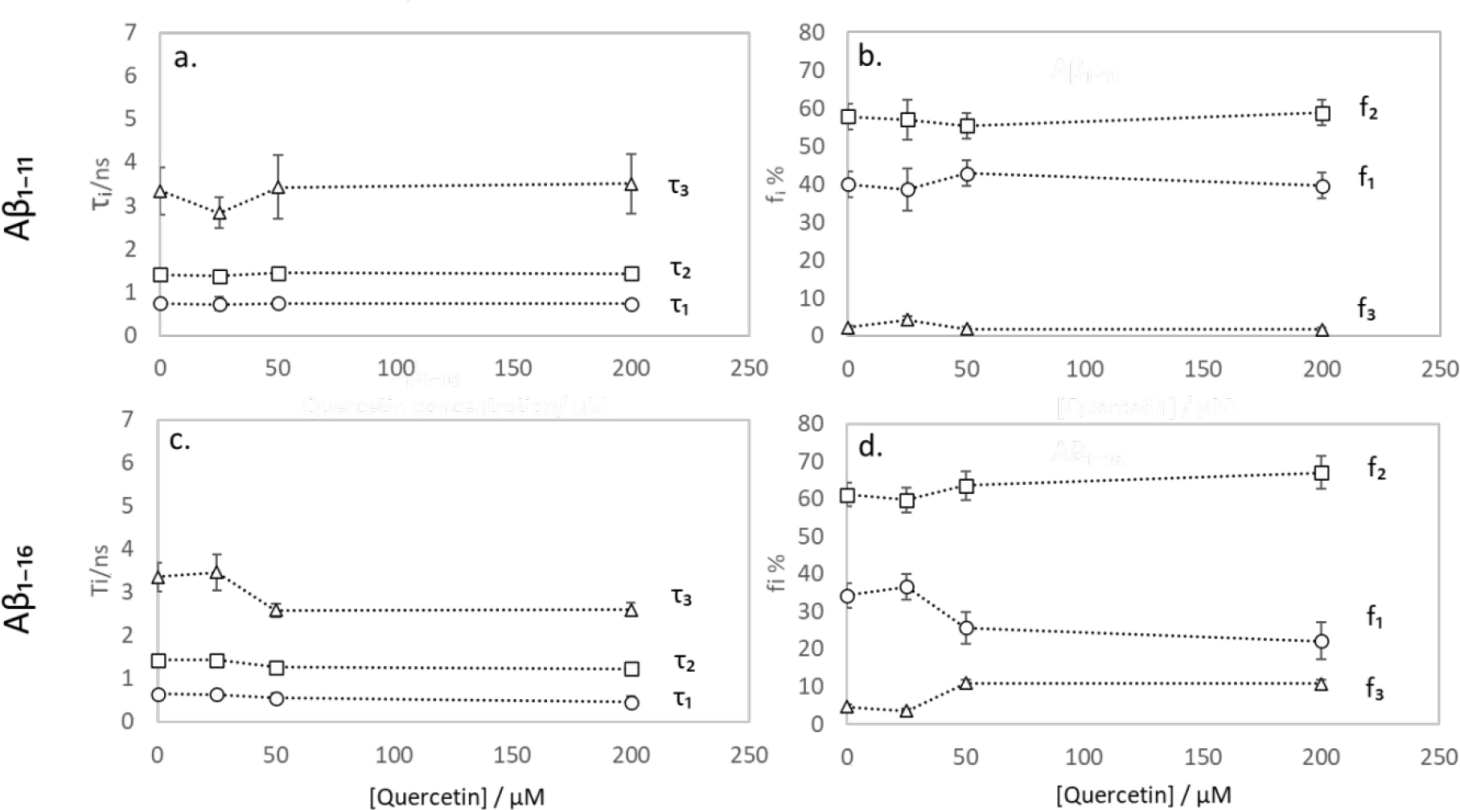
Parameters obtained from fitting Tyr’s fluorescence decay in a 50 μM Aβ_1–11_ (a, b) and Aβ_1–16_ (c, d) solution at emission wavelength 316 nm to a three-exponential decay model plotted against quercetin concentration: Tyr fluorescence decay times τ_1_, τ_2_ and τ_3_ in Aβ_1–11_ (a) and Aβ_1–16_ (c), percentage contributions *f*_1_, *f*_2_, and *f*_3_ in Aβ_1–11_ (b) and Aβ_1–16_ (d). Error bars represent 3 × standard deviation.

Fluorescence intensity decays of both Aβ_1–11_ and Aβ_1–16_ were best fitted to a 3-exponential model (Figure 3). The decay times *τ*_1_, *τ*_2_, and *τ*_3_ and their *respective* percentage contributions *f*_1_, *f*_2_, and *f*_3_ for Tyr in *both the* Aβ_1–11_ and Aβ_1–16_ samples showed no significant change with an increase in quercetin concentration. This indicates that the presence of quercetin does not impact Tyr_10_ fluorescence kinetics directly and confirms that the decrease in Aβ_1–11_ and Aβ_1–16_ emission intensity is due to the inner filter effect. To avoid this, quercetin in Aβ_1–40_ samples was kept at concentrations not exceeding 50 μM. Figure S4 also shows that quercetin (at 50 μM) does not have a direct effect on the intensity of Aβ_1–40_ emission (after 10 min) but surely has an effect on the aggregation process (after 24–142 h).

### Aβ_1–40_ and Aβ_1–40_-Quercetin Samples

**Figure 4:**
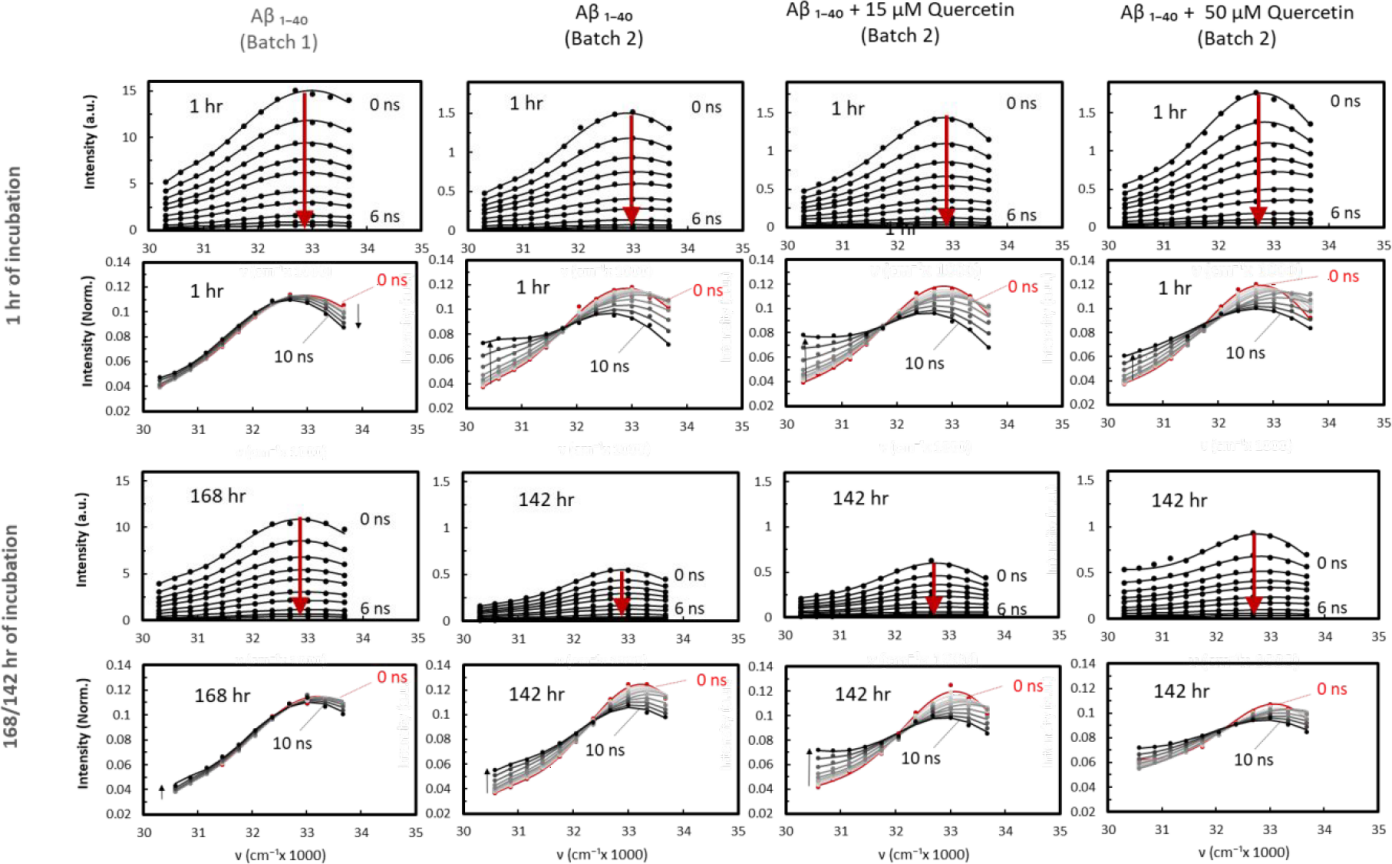
Time-resolved emission spectra (TRES) obtained for 50 μM Aβ_1–40_ in HEPES buffer (pH 7.4) in the absence (batch 1 and 2) and presence of two different concentrations of quercetin 15 and 50 μM (batch 2) after 1 h of incubation and 142/168 h of incubation. The solid lines represent the two-Toptygin-type function fits.

TRES were obtained for a sample of Aβ_1–40_ in the absence of quercetin and in the presence of 15 and 50 μM of quercetin. Figure 4 shows TRES measured after 1 h and 142 h of incubation at 37 °C. The measurements obtained for the no-quercetin sample were first compared with the results obtained in our previous Aβ_1–40_ TRES studies (batch 1).^24^ The differences between the results for batch 1 and our current sample (batch 2) suggest that, in spite of applying the same sample preparation procedures in both cases (treatment by HFIP), batch 2 contains considerably more preformed aggregates. This demonstrates that the individual batches of even freshly purchased Aβ_1–40_ material may not be identical in terms of the degree of aggregation and that the measurement of the “blank” sample needs to precede the measurements for the studied process and should be used as a reference. This also demonstrates the high sensitivity of our approach in establishing the stage of aggregation.

**Figure 5:**
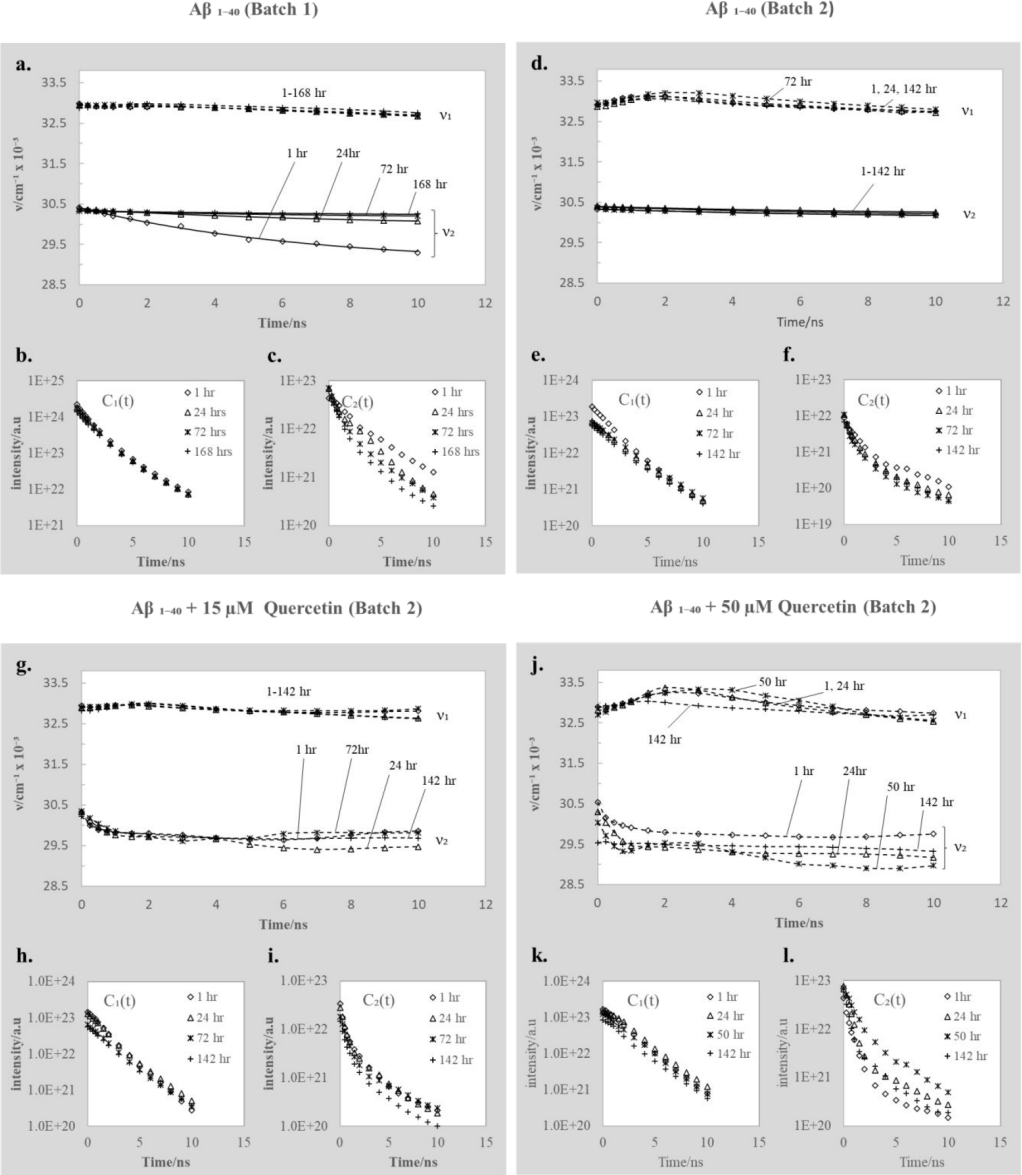
Peak positions ν_1_(*t*) and ν_2_(*t*) obtained from fitting Aβ_1–40_ TRES to eq 3, plotted against time in nanoseconds at different stages of aggregation for samples without (a, d) and with (g, j) quercetin. The fluorescence intensity decay of each component *C*_1_(*t*) and *C*_2_(*t*) obtained from fitting eq 3 to the experimental TRES data for the sample without (b, c, e, f) and with (h, i, k, l) quercetin.

TRES of Aβ_1–40_ in all samples were sufficiently represented by two peaks (*N* = 2) of the Toptygin-type function (3). The parameters recovered for all samples are plotted in Figure 5. We attribute the spectral peak values ν_1_(*t*) to monomers and/or small oligomers, and ν_2_(*t*) to larger oligomers.^24^

Note that the initial (*t* = 0) positions of all monomer peaks ν_1_(*t*) are located at ∼33000 cm^–1^, and the initial (*t* = 0) positions of all oligomer peaks ν_2_(*t*) are located at ∼30500 cm^–1^. These values are obtained mathematically when calculating the TRES data from the experimentally measured fluorescence decay, and importantly, they do not represent the energies of spectral maxima of the initial nonequilibrium Franck–Condon state of the excited tyrosine. Their shifts over 10 ns after excitation are highly influenced by the presence of Aβ_1–40_ aggregates, the concentration of quercetin, and the age of the sample. The fluorescence intensity decay of each component is represented by the *C*_1_(*t*) and *C*_2_(*t*) functions obtained from fitting eq 3 to the experimental TRES data.

Here we discuss separately the results obtained in all four samples.

#### Free Ab_1–40_ (Batch 1)

The position of the peak ν_1_(*t*) exhibits a very slow drop (Figure 5a). Indeed, in this case the Aβ_1–40_ peptides are small in size and exposed to water molecules; thus, the dielectric relaxation process is fast and almost completed before fluorescence occurs. The position of ν_2_(*t*) at 1 h after sample preparation, in the Aβ_1–40_ sample free from aggregates (Figure 5a), shifts exponentially from ν_2_(0) ≈ 30500 cm^–1^ to ν_2_(∞) ≈ 29000 cm^–1^ with the dielectric relaxation time τ_R_ = 6.7 ns. As the sample ages, the relaxation time τ_R_ increases, and the shift toward the red is reduced, due to the expected gradual growth in the size of the aggregates. We also note that the decay rate *C*_1_(*t*) is not affected by the age of the sample, which is natural considering that *C*_1_(*t*) represents monomers (Figure 5b), while the decay rates of the oligomers *C*_2_(*t*) (Figure 5c) are increasing with the age of the sample, reflecting more efficient quenching in larger aggregates.

#### Free Ab_1–40_ (Batch 2)

In the sample of partially aggregated peptides, the position of the peak ν_1_(*t*) (Figure 5d) exhibits an initial shift toward higher wavenumbers followed by a shift toward lower wavenumbers. This behavior (also observed for ν_1_(*t*) in the presence of quercetin, see Figure 5g,j) is unusual for the dielectric relaxation, and we believe that the ν_1_(*t*) curves represent more than one form, most likely both monomers and very small oligomers, exhibiting slightly different fluorescence decay rates and relaxation times. The ν_2_(*t*) (Figure 5d) exhibits a relatively small exponential shift with dielectric relaxation time τ_R_ = 8.7 after 1 h of incubation. Again, the relaxation time τ_R_ increases with time of incubation, due to the growth of aggregates. Note that the behavior of *v*_2_(*t*) in Figure 5d is similar to that observed in Figure 5a at later times, suggesting that the processes in batch 2 were the same as in batch 1, but their monitoring started at the later stage of aggregation. Moreover, the decays *C*_1_(*t*) and *C*_2_(*t*) exhibit a change within 24 h only and then remain almost constant at later times (Figure 5e,f), which supports the above suggestion.

#### Ab_1–40_ + 15 μM Quercetin (Batch 2)

Adding 15 μM quercetin to the sample of Aβ_1–40_ with existing aggregates (Figure 5g) has significantly changed the ν_2_(*t*) behavior; it clearly shows a dielectric relaxation shift during the first 2 ns after excitation and then becomes constant. The curves are almost the same, when measured after 24, 72, and 142 h of incubation, suggesting that the aggregates formed might have reached stability much earlier than the free Aβ_1–40_ aggregates. The presence of the solvatochromic shifts, which are not observed in the free Aβ_1–40_ (batch 2) sample, suggests that quercetin forms complexes with Aβ_1–40_ peptides, and they are smaller than aggregates formed in the free Aβ_1–40_ samples at the same point in time during the aggregation process. Also, their formation prevents any further aggregation. The decays *C*_1_(*t*) and *C*_2_(*t*) are similar to those observed for free Ab_1–40_ (batch 2) (Figure 5h,i).

#### Ab_1–40_ + 50 μM Quercetin (Batch 2)

At a higher quercetin concentration, the ν_2_(*t*) dependence is modified further (Figure 5j). The evolution in ν_2_(*t*) is nonexponential and is sensitive to the time of incubation. Clearly the model assuming two components is even more inadequate for the increased presence of quercetin. A more likely explanation is a variety of aggregates of different sizes, each exhibiting a distinct dielectric relaxation and fluorescence lifetime. The appearance of the solvatochromic shift suggests again that the Aβ_1–40_-quercetin aggregates are smaller than the free Aβ_1–40_ aggregates at the same point in time. The lack of the shift in the position of ν_2_(*t*) observed after 142 h of incubation suggests that the dielectric relaxation is finally blocked due to restricted rotational freedom of the environment surrounding tyrosine in large aggregates. At 50 μM of quercetin, the intensity decay of the first component *C*_1_(*t*) shows no significant changes, whereas *C*_2_(*t*) appears to be very sensitive to the age of the sample. The mean decay rate is not changing monotonically, which is likely to be caused by the variety of aggregate forms of different lifetimes and quantum yields, whose presence is changing during the aggregation process.

**Figure 6:**
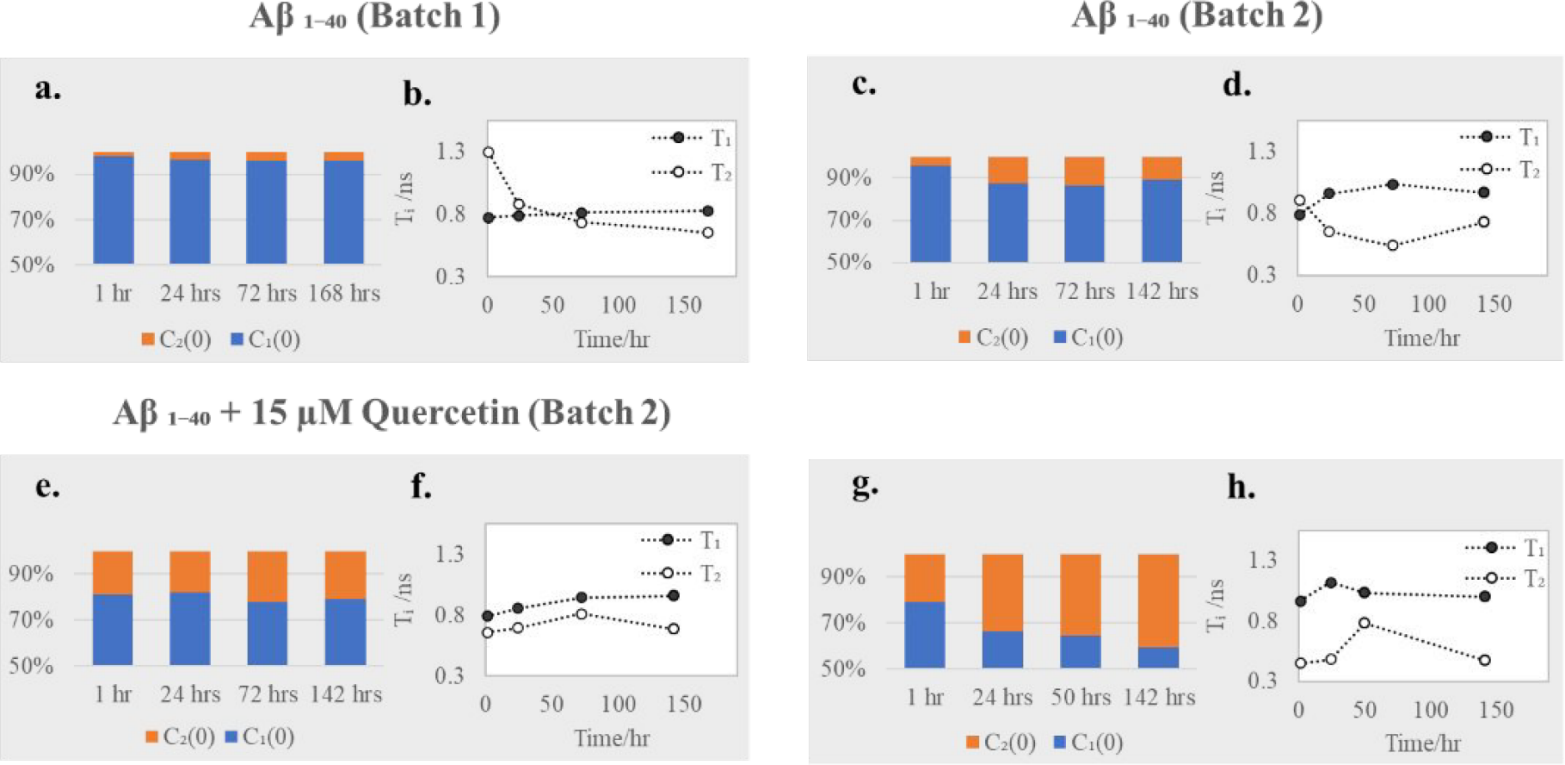
Initial percentage contribution of fluorescence intensity of the two emitting species, monomers *C*_1_(0) and oligomers *C*_2_(0) attained from fitting eq 3 to the measured TRES of Aβ_1–40_ (batch 1) (a) and Aβ_1–40_ (batch 2) in the absence (c) and the presence of two concentrations of quercetin 15 μM (e) and 50 μM (g). The characteristic fluorescence lifetimes of monomers *T*_1_ and oligomers *T*_2_ for Aβ_1–40_ (batch 1) (b) and Aβ_1–40_ (batch 2) in the absence (d) and presence of quercetin 15 μM (f) and 50 μM (h).

Further information on the effects of quercetin can be drawn out from the initial percentage contributions of fluorescence intensities of the two emitting species, monomers *C*_1_(0) and oligomers *C*_2_(0), and the fitted mean lifetimes *T*_1_ and *T*_2_, calculated from *C*_1_(*t*) and *C*_2_(*t*) curves (Figure 6).

After 1 h of incubation, 98% of fluorescence emission in batch 1 comes from monomers (Figure 6a), and as the sample ages, the percentage contribution of monomer *C*_1_(0) slightly decreases to about 95% indicating that monomers are aggregating and forming larger structures. *C*_1_(*t*) decays with a characteristic lifetime *T*_1_ ≈ 0.8 ns regardless of the sample’s actual age (Figure 6b), which is consistent with *C*_1_(*t*) representing monomers. The characteristic lifetime *T*_2_ of the decay *C*_2_(*t*) associated with oligomers decreases from 1.3 to 0.7 ns as the Aβ_1–40_ sample ages (Figure 6b), which may be explained by more intense quenching of fluorescence in larger aggregates.

In initially partially aggregated batch 2, the percentage contribution of monomers to fluorescence emission is reduced to about 95% after 1 h of incubation (Figure 6c) and continues to decrease with time of incubation until it reaches about 89% after 142 h. *T*_1_ increases with time of incubation from 0.8 to about 1 ns (Figure 6d), while the initial value of *T*_2_ is reduced to about 0.9 ns and continues to decrease over time (Figure 6d). This is an indication that the components *C*_1_(*t*) and *C*_2_(*t*) represent more than only two structures of distinct fluorescence lifetimes, which is consistent with the already mentioned unusual behavior of ν_1_(t) (Figure 5d).

Adding 15 μM of quercetin to the Aβ_1–40_ sample reduces the initial contribution of monomers to about 80% after 1 h of incubation (Figure 6e). Increasing the quercetin concentration to 50 μM (Figure 6g) demonstrated further reduction in the contribution of monomers. In the presence of quercetin, both at the level of 15 and 50 μM, the *T*_1_ behavior is similar (Figures.6f,h), suggesting that, as expected, the quercetin presence in the sample does not affect the monomers and smallest oligomers. Simultaneously, the initial value of *T*_2_ is further reduced to 0.7 and 0.5 ns at concentrations 15 and 50 μM, respectively, due to increased Tyr quenching in larger aggregates.

To summarize the TRES analysis, parameters obtained from fitting eq 3 to the data collectively suggest that aggregation progresses differently in the presence of quercetin due to the formation of Aβ-quercetin complexes, which are likely to prevent further Aβ aggregation. The Aβ-quercetin complexes are not only different than aggregates without quercetin, but also are smaller, because formation of the big structures (which prevent dielectric relaxation) is hindered.

**Figure 7:**
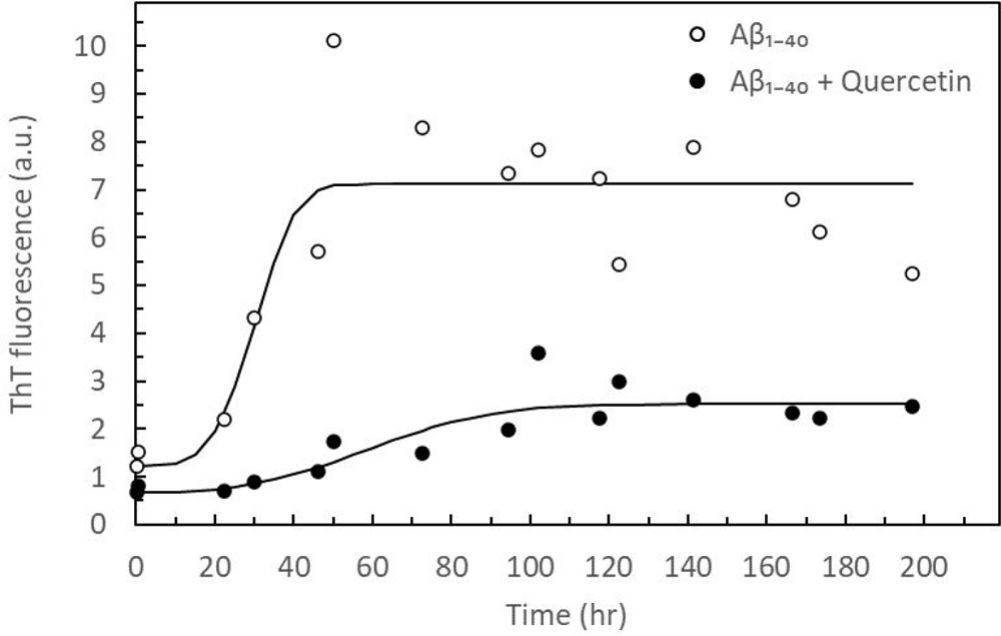
Time-dependent ThT fluorescence intensities measured at the peak ThT emission (484 nm) for 50 μM Aβ_1–40_ (batch 2) in the absence (○) and presence of 50 μM quercetin (●). The solid line is a guide to the eye.

To examine further the stages of Aβ_1–40_ aggregation in the presence of quercetin, the process was monitored by the ThT binding assay in the absence and presence of 50 μM quercetin (Figure 7). Results show that Aβ_1–40_ starts forming β-sheet-rich aggregates after ∼24 h. A relatively rapid aggregation phase begins and, after ∼50 h, is followed by the saturation phase. Adding quercetin dramatically reduces the increase in ThT fluorescence, which can be explained by a much lower presence of β-sheet structures (thus prevented Aβ_1–40_ aggregation), providing that the potential quenching of ThT fluorescence by quercetin can be eliminated. Figure S5 shows that adding quercetin to a ThT solution does not reduce the ThT fluorescence; thus, no ThT quenching is observed. Therefore, the drop in ThT fluorescence in an Aβ_1–40_-containing sample confirms that quercetin not only alters the aggregation pathways but also hinders formation of β-sheets.

## Conclusion

TRES measurements show a clear difference between the two batches of Aβ_1–40_. We attribute this difference to the presence of preformed aggregates in the second batch. The presence of such aggregates affects the lag time for fibril formation. It may also have an impact on quercetin’s ability to alter the aggregation pathway.

TRES data have also shown that the kinetics of Aβ_1–40_ aggregation in the presence of quercetin produces different fluorescent aggregates, as indicated by different changes in both peaks ν_1_(*t*) and ν_2_(*t*), with *v*_2_(*t*) showing dielectric relaxation. More interestingly, the fluorescence intensity contribution of the component *C*_2_(*t*) at *t* = 0 increases with quercetin concentration. This suggests an increase in the number oligomers, rather than an increase in their size. This is consistent with the ThT binding assay confirming the lack of β-sheet structures. Thus, quercetin experiments show early formation of the Aβ-quercetin complexes, which seem to inhibit further Aβ aggregation. This fact, combined with quercetin being a natural nontoxic substance capable of crossing the blood–brain barrier, makes it a potential nutrient helping to prevent the onset and the development of Alzheimer’s disease.

## Supporting Information Available

Time-correlated single photon counting (TCSPC) measurements, TRES of Aβ_1–40_ at several stages of aggregation, absorption and emission spectra for tyrosine and quercetin, and emission spectra of Aβ_1–40_ (PDF)
