# Supplementary material for: Impact of the Flavonoid Quercetin on β-Amyloid Aggregation Revealed by Intrinsic Fluorescence
Source: J Phys Chem B. 2022 Sep 19;126(38):7229–37. doi: 10.1021/acs.jpcb.2c02763 (PMC9527748; doi:10.1021/acs.jpcb.2c02763)
Supplement: Supplementary file 1 — jp2c02763_si_001.pdf [file jp2c02763_si_001.pdf]

Impact of the flavonoid quercetin on beta-amyloid aggregation revealed by intrinsic fluorescence.

SUPPLEMENTARY INFORMATION

Abeer Alghamdi[a], David J.S. Birch[a] , Vladislav Vyshemirsky[b], Olaf J. Rolinski[a,*]

[a]Photophysics Group, Centre for Molecular Nanometrology, Department of Physics, Scottish Universities Physics Alliance, University of Strathclyde, 107 Rottenrow East, Glasgow G4 0NG, UK; Email: o.j.rolinski@strath.ec.uk

[b]School of Mathematics and Statistics, University of Glasgow, Glasgow, G12 8QQ, UK

1. Time-correlated single photon counting (TCSPC)

TCSPC measurements were conducted on a Horiba Scientific DeltaFlex fluorometer (HORIBA Jobin Yvon IBH Ltd,Glasgow,UK). The system was equipped with Seya-Namioka monochromators for excitation and emission. The excitation source used was a HORIBA NanoLED with a centre wavelength of 279 nm, pulse duration of 50 ps and a repetition rate of 1 MHz [31].

A series of 12 fluorescence decay curves were collected at the emission wavelengths between 297 and 330 nm at 3 nm increments. Fig. S1 shows an example of three fluorescence decays

measured at different stages of aggregation (1, 48 and 168 hrs) for free Aβ and Aβ with Quercetin

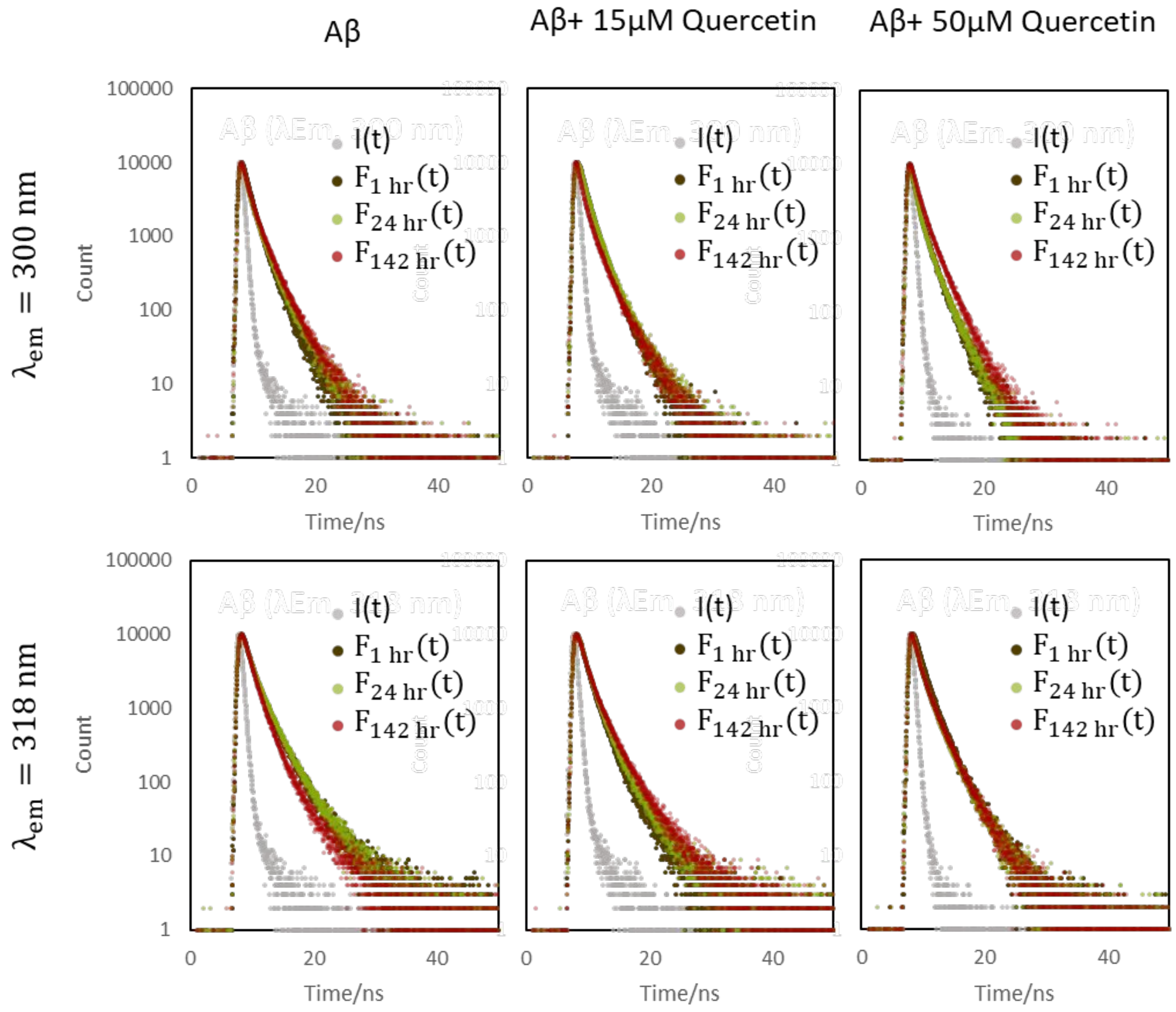

Figure S1. *Fluorescence decay data of $A\beta_{1-40}$ at two detection wavelengths 300 and 318 nm together with the excitation pulse I(t) obtained at three different stages of aggregation 1hr ($F_{1hr}(t)$) ,48 hrs ($F_{24hr}(t)$) and 142hrs ($F_{142}(t)$) for free Aβ and Aβ with 15 μM and 50 μM of Quercetin.*

2. TRES

TRES were obtained for $A\beta_{1\text{-}40}$ at several stages of aggregation, namely 1, 24, 50/72 and 140/168 hrs after sample preparation (the number indicates the age of the sample when the measurement at the first wavelength has been started). From the data obtained for each stage of aggregation, 14 TRES were calculated at different times after excitation. Fig. S2 shows

the TRES of samples in the absence (batch1 and batch2) and presence of 15, 50 mM of quercetin (Batch 2) at different stages of aggregation (i.e.1, 24, 50/72 and 142/168 hours)

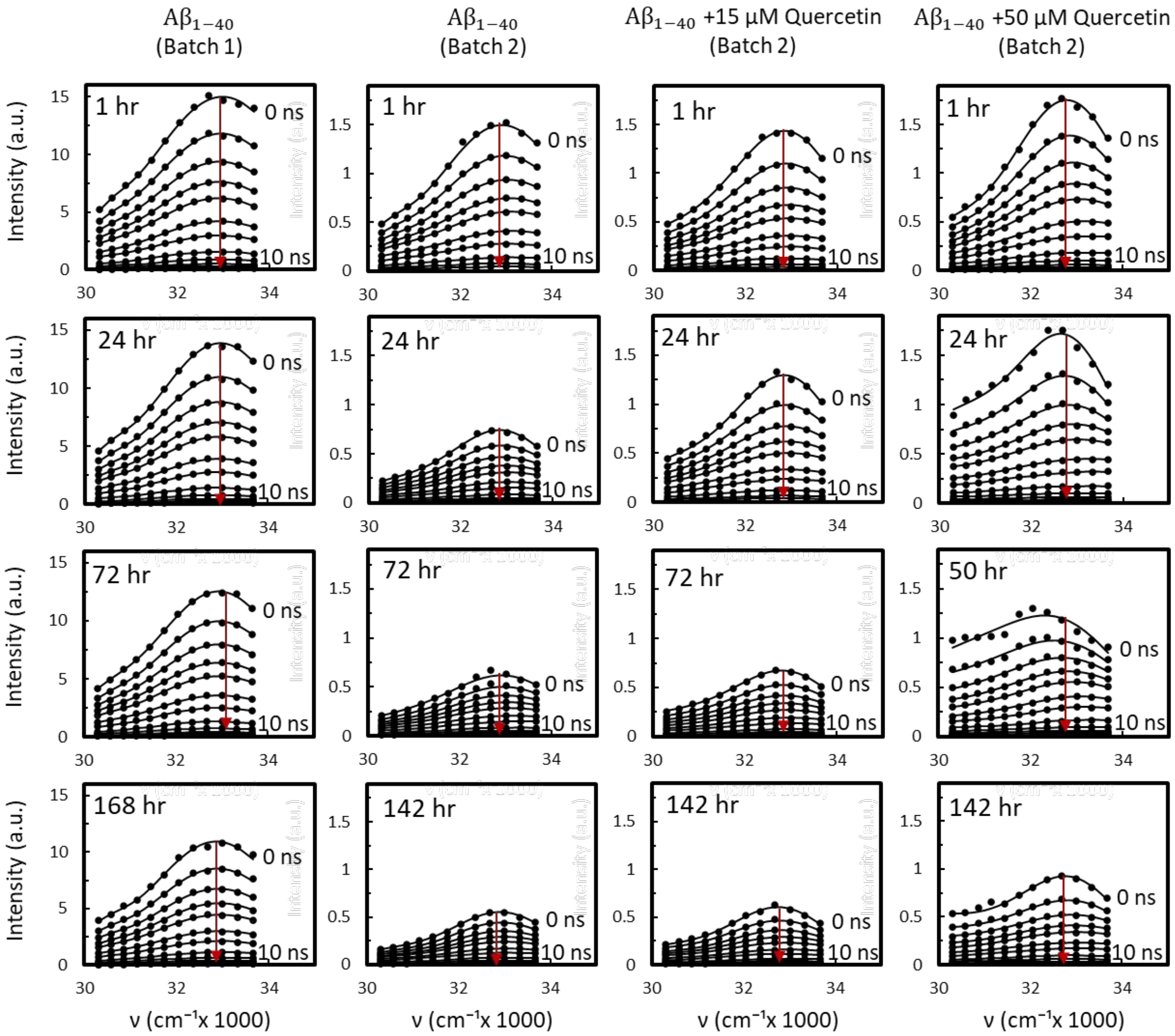

Figure S2. *Time-resolved emission spectra (TRES) obtained for 50 μM Aβ₁₋₄₀ in HEPES buffer (pH 7.4) in the absence (batch 1 and 2) and presence of two different concentrations of quercetin 15 and 50 μM (batch 2) after 1hr of incubation and 142/168 hr of incubation. The solid lines represent the two-Toptygin type functions fits.*

3. Absorption and emission spectra for Tyrosine and Quercetin

UV-Vis absorbance measurements were carried out using a Perkin Elmer 25. All measurements were carried out at a concentration of 50 μM.

Steady-state fluorescence spectra of Tyrosine and quercetin were obtained using a Fluorolog-3 spectrofluorimeter. The excitation and emission monochromators were set at 5-nm slit widths. Tyr was excited at 279 nm and emission spectrum was recorded at 290 to 500 nm in

1 nm increments. Quercetin was excited at 380 nm and emission spectrum was recorded at 350 to 740 nm in 1 nm increments.

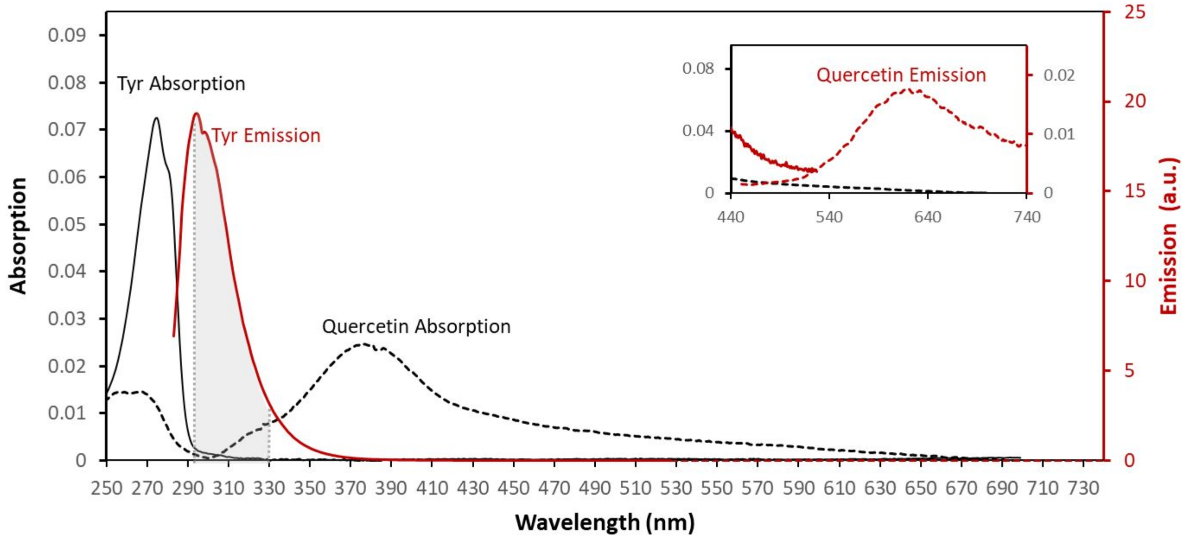

Figure S3. *Absorption (black) and fluorescence emission (red) spectra of 50 µM Tyr (solid lines) and 50 µM Quercetin (dotted lines). Inset shows an enlargement of the quercetin emission spectra.*

4. Emission spectra of Aβ$_{1-40}$

Steady-state fluorescence spectra of ThT and Tyrosine in Aβ$_{1-40}$ were obtained using a Fluorolog-3 spectrofluorimeter. The excitation and emission monochromators were set at 5-nm slit widths. Tyr was excited at 279 nm and emission spectra were recorded at 290 to 500 nm in 1 nm increments. Measurements were repeated for the Aβ$_{1-40}$ sample at different times: 10 min, 24 hr, 72 hr and 140 hr after sample preparation.

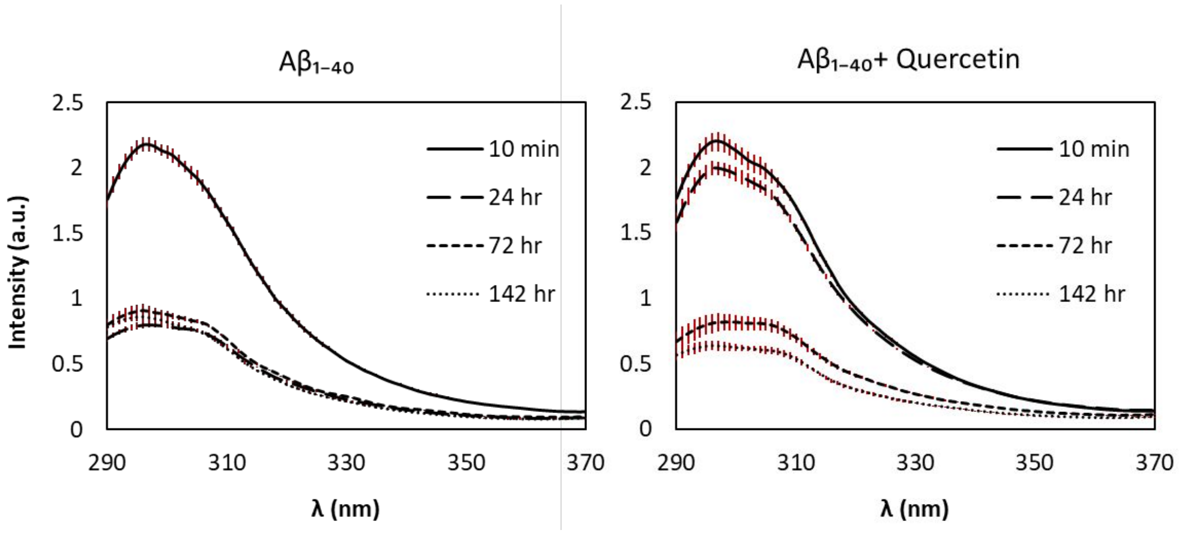

Figure S4. *Fluorescence emission spectra of Aβ$_{1-40}$ (50 μM, pH 7.4) in the absence (left) and presence (right) of 50 μM quercetin obtained at different moments in time 10 min (solid line), 24 hr (long-dashed line), 72 hr (short-dashed line) and 142 hr (dotted line)*

ThT was excited at 450 nm, and the emission spectra were recorded from 465 to 600 nm over a 200 hrs time period.

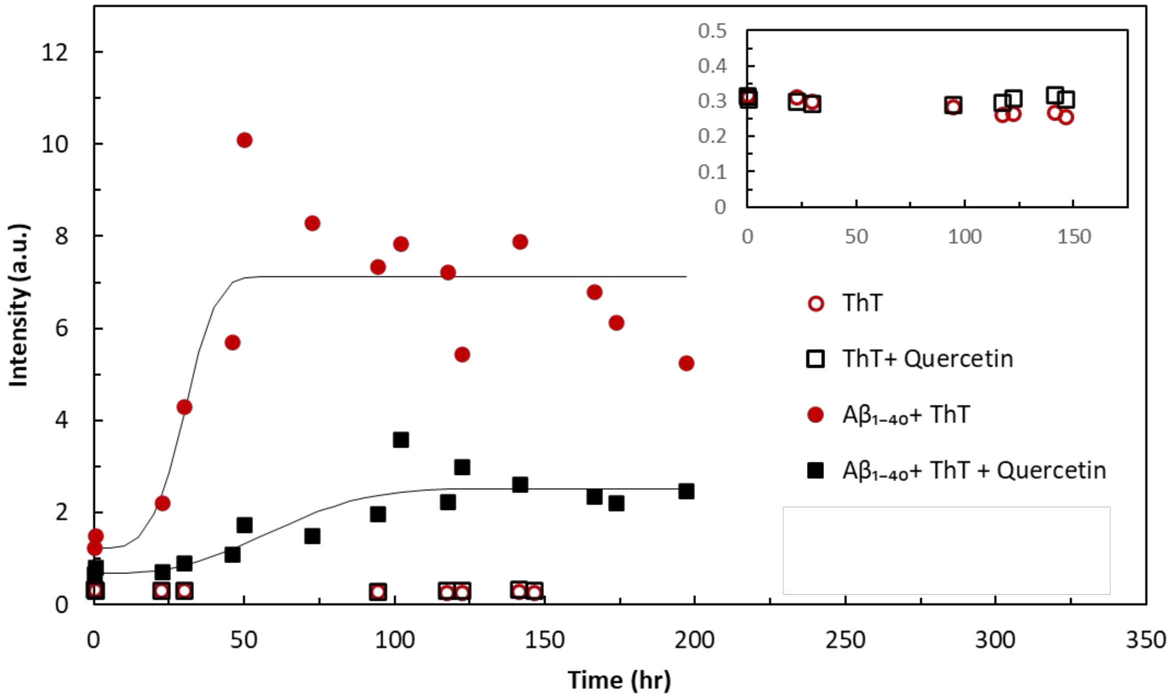

Figure S5. *Time-dependent ThT fluorescence intensities measured at the peak of ThT emission (484 nm) for 50 μM Aβ$_{1-40}$ (Batch 2) in the absence (●) and presence of 50 μM Quercetin (■), Th T alone (○) and Th T with Quercetin (□). The solid line is a guide to the eye. Inset shows plots with enlarged scale.*
